# Be Healthy in Pregnancy (BHIP): A Randomized Controlled Trial of Nutrition and Exercise Intervention from Early Pregnancy to Achieve Recommended Gestational Weight Gain

**DOI:** 10.3390/nu14040810

**Published:** 2022-02-15

**Authors:** Stephanie A. Atkinson, Atherai Maran, Kendra Dempsey, Maude Perreault, Thuva Vanniyasingam, Stuart M. Phillips, Eileen K. Hutton, Michelle F. Mottola, Olive Wahoush, Feng Xie, Lehana Thabane

**Affiliations:** 1 Department of Pediatrics, McMaster University, Hamilton, ON L8S 4K1, Canada; atheraimaran@gmail.com (A.M.); kendradempsey@gmail.com (K.D.); perreault.maude@gmail.com (M.P.); 2 Department of Health Research Methods, Evidence, and Impact, McMaster University, Hamilton, ON L8S 4K1, Canada; thuva.vanni@gmail.com (T.V.); thabanl@mcmaster.ca (L.T.); 3 Department of Kinesiology, McMaster University, Hamilton, ON L8S 4K1, Canada; phillis@mcmaster.ca; 4 Midwifery Research Centre, McMaster University, Hamilton, ON L8S 4K1, Canada; huttone@mcmaster.ca; 5 Department of Anatomy & Cell Biology, School of Kinesiology, University of Western Ontario, London, ON N6A 3K7, Canada; mmottola@uwo.ca; 6 School of Nursing, McMaster University, Hamilton, ON L8S 4K1, Canada; wahousho@mcmaster.ca; 7 Centre for Health Economics and Policy Analysis, McMaster University, Hamilton, ON L8S 4K1, Canada; fengxie@mcmaster.ca

**Keywords:** nutrition, exercise, randomized controlled trial, gestational weight gain, pregnancy, infancy, developmental origins of health and disease, protein, dairy foods

## Abstract

A randomized two-arm prospective superiority trial tested the efficacy of a novel structured and monitored nutrition (bi-weekly counselling for individualized energy and high dairy protein diet) and exercise program (walking goal of 10,000 steps/day) (intervention) compared to usual care (control) in pregnant women to achieve gestational weight gain (GWG) within current recommendations. Women recruited in communities in southern Ontario, Canada were randomized at 12–17 weeks gestation with stratification by site and pre-pregnancy BMI to intervention (*n* = 119) or control (*n* = 122). The primary outcome was the proportion of women who achieved GWG within the Institute of Medicine recommendations. Although the intervention compared to control group was more likely to achieve GWG within recommendations (OR = 1.51; 95% CI (0.81, 2.80)) and total GWG was lower by 1.45 kg (95% CI: (−11.9, 8.88)) neither reached statistical significance. The intervention group achieved significantly higher protein intake at 26–28 week (mean difference (MD); 15.0 g/day; 95% CI (8.1, 21.9)) and 36–38 week gestation (MD = 15.2 g/day; 95% CI (9.4, 21.1)) and higher healthy diet scores (22.5 ± 6.9 vs. 18.7 ± 8.5, *p* < 0.005) but step counts were similar averaging 6335 steps/day. Pregnancy and infant birth outcomes were similar between groups. While the structured and monitored nutrition with counselling improved diet quality and protein intake and may have benefited GWG, the exercise goal of 10,000 steps/day was unachievable. The results can inform future recommendations for diet and physical activity in pregnancy.

## 1. Introduction

Despite the available guidelines on gestational weight gain (GWG) from the Institute of Medicine since 2009 [1], excess GWG is highly prevalent in women across all pre-pregnancy body mass index (pBMI) categories in both Canada and the United States [2,3]. In observational studies, excessive GWG and pregravid obesity were associated with greater risk of adverse perinatal outcomes [4,5,6,7,8] and risk of infant macrosomia or large-for-gestational age rises with maternal pregravid obesity and excessive GWG [9,10,11,12].

The experimental intervention in the Be Healthy in Pregnancy (BHIP) randomized controlled trial (RCT) [13] was grounded in a synthesis of existing systematic reviews of RCTs with varying intervention strategies that suggested the most convincing approach to achieve reduction in GWG was a combination of physical activity and individualized diet counselling, preferably in combination with weight monitoring [14,15,16].

The primary objective of the BHIP study was to determine if introducing a structured and monitored nutrition and exercise program (intervention) in early pregnancy compared with usual prenatal care (control) increased the likelihood of attaining GWG within the IOM guidelines (outcome) over the pregnancy period. We hypothesized that the experimental combined structured and monitored Nutrition + Exercise intervention compared with usual care would increase the percentage of pregnant women who achieved a GWG within IOM recommendations overall and for each pre-pregnancy BMI category and site. Other pre-specified outcomes included comparison of pregnancy and birth outcomes and maternal metabolic health between intervention and control groups.

## 2. Materials and Methods

### 2.1. Study Design, Data Collection, and Ethical Aspects

The BHIP study was a two-arm, two-site prospective superiority trial with 1:1 allocation ratio to either the intervention group (i.e., Nutrition + Exercise with counselling and monitoring) or the control group (usual care as per Health Canada recommendations) from early pregnancy to birth. Ethics approval was obtained from the Research Ethics Boards of Hamilton Health Sciences (REB Project#12-469), Western University, London (HSREB 103272), and Joseph Brant Hospital, Burlington (JBH 000-018-14) in Ontario, Canada. The trial was registered in 2012 [17].

### 2.2. Recruitment, Randomization, and Followup

Healthy pregnant women who represented all pBMI categories except extreme obesity (>40 kg/m^2^) were recruited from community health care clinics in Hamilton, Burlington and London, Ontario between 12 and 17 weeks of gestation from January 2013 to April 2018 as previously detailed in the study protocol paper [1]. Eligibility criteria were screened at the first contact and the baseline visit [13].

Block randomization to the two study arms was conducted at the second visit to the study center and was stratified by study site and pBMI category used by IOM [1] as detailed previously [13]. The study was open-label with blinded endpoints due to the nature of the intervention as detailed [13].

All participants received standard care delivered by their health care practitioner (physician or midwife) during pregnancy. After enrolment, all participants received counselling and hard copies of the advice from Health Canada at the time of the trial as published online [18,19,20]. The primary caretaker of each participant also received mailed copies of the same materials. A $25 Canadian grocery gift card was available for all participants on three occasions when dietary and accelerometry data were collected.

#### 2.2.1. Intervention

The multi-component intervention strategy included the specific active intervention of a high protein dairy diet and individualized energy intake found effective in our previous two studies in non-pregnant women [21,22] and proven reasonable by our qualitative testing for feasibility [23]. In-person counselling occurred weekly or biweekly from early in the second trimester (14–17 weeks gestation) until the end of pregnancy as detailed [13]. The study nutritionist counselled the participants on their individual dietary goals, providing recipes and diet plans suited to meet energy requirements. The individualized goals were based on providing 25% of their energy intake from protein of which 50% of the protein was derived from dairy foods as approximately 4–6 servings daily of low-fat dairy, such as milk, cottage cheese, and yogurt according to each participant’s preference. The diet plan was set to each individual’s calculated Estimated Energy Requirements (EER) using the equations in the Dietary Reference Intake report for normal and overweight pregnant women based on their size, age, and physical activity levels, with additional energy goals added as pregnancy progressed [24]. Several strategies to promote adherence to the diet intervention were included such as provision of low-fat dairy foods as per participant preference as detailed previously [13]. For the walking-based exercise program, participants first completed a validated activity questionnaire which was reviewed by a certified exercise physiologist to assess participants’ activity levels at baseline. Participants were encouraged to follow guidelines from the PARmed-X for Pregnancy [22], walking 3–4 times each week for 25 min a session, increasing their walking time by 2 min a week until they reached 40 min. At each study visit, the research assistant walked with the participant for 30 to 40 min around campus or within the medical center/hospital during inclement weather. The goal was to walk 10,000 steps/day; any daily form of physical activity counted towards the step count, including walking sessions at study visits accompanied by research staff and daily habitual activities, and were measured through use of a pedometer with records kept on pedometer logs that were reviewed at each study visit [13].

#### 2.2.2. Usual Care

The control group received usual care provided by their primary care provider and only returned to the study site for outcome measurements at 26–28 and 36–38 weeks of gestation. As a retention strategy, control participants were invited to join a focus group in the second trimester led by a midwife, with topics including pain relief options during labor and breastfeeding techniques.

### 2.3. Outcomes and Measurements

The primary outcome was the proportion of women who were within the appropriate GWG for pBMI category using the IOM 2009 recommendations for total GWG and GWG per week over the second and third trimesters of pregnancy [1]. Body weight was measured by trained research assistants using a Tanita Body Composition Analyzer BF-350 scale (Tanita, Arlington Heights, IL, USA) and GWG calculated as previously described [13].

Demographic information was obtained at baseline using a standard questionnaire and screening for depression score used the Edinburgh Depression Scale [25]. Diet assessment was completed at 12–17, 26–28, and 36–38 weeks gestation and included a 21-item food frequency questionnaire (FFQ) adapted from the validated PrimeScreen FFQ [26] to derive a score for healthy dietary practices and a 3-day food intake record to determine protein and energy intakes [13]. Physical activity and exercise behaviors were assessed during the same 3 days using a SenseWear armband tri-axis accelerometer (Model MF-SW:BodyMedia Inc., Pittsburgh, PA, USA) and analyzed to obtain step count, energy expenditure, and minutes of activity level using SenseWear Professional 8.1 Software (BodyMedia Inc., Pittsburgh, PA, USA).

Obstetrical outcomes obtained from medical charts or by self-report by the participants included delivery mode, diagnosis of gestational diabetes mellitus (GDM defined as blood glucose of >7.2 mmol/L 2 h after a 75 g oral glucose tolerance test), and pre-eclampsia. At 12–17 and 36–38 weeks gestation, blood pressure was measured and fasting blood samples obtained for profiles of lipids, glucose, C-reactive protein, and hormones leptin and adiponectin by methods previously detailed [13]. At these two time points and at 26–28 weeks gestation, skinfold thickness was measured in triplicate (Harpenden Skinfold Caliper, Baty International, West Sussex, UK) by trained staff on the right-hand side of the body at four sites—subscapular, triceps, biceps, and supra-iliac crest for which triplicate values were averaged and sum of skinfold thickness (SFT) calculated. Percent body fat was estimated from leg-to-leg body impedance by using the Tanita Body Composition Analyzer BF-350 scale (Tanita, Arlington Heights, IL, USA).

Pre-specified infant outcomes extracted from medical charts included gestational age, birth weights which were categorized as small, appropriate, or large for gestational age [27], length, head circumference, 1- and 5-min Apgar scores, complications related to birth, and feeding practices at birth.

### 2.4. Sample Size and Statistical Analysis

The proposed sample size of 155 participants per treatment group was based on the test of the null hypothesis that the percentages of women with GWG within IOM guidelines in the two populations (intervention and control) are equal as detailed in our research design paper [13]. We revised our target sample size based on our a priori calculation to a sample size of 111 per group corresponding to 30% of women in the treatment group having a GWG exceeding the IOM recommendations compared to 65% in the control group. With the revised sample size (i.e., assuming a 1:1 allocation ratio), the study would have power of 80% to yield a statistically significant result assuming a binomial distribution (using an intention-to-treat principle for the analysis) of the difference between percentages of women with GWG within IOM guidelines at alpha = 0.05.

Data were entered into an online Research Electronic Data Capture (REDCap) service hosted at McMaster University) [13]. Descriptive statistics of participant baselines characteristics and outcome variables (both primary and secondary) were reported as detailed previously [13]. The primary analysis for all outcomes was intention to treat (ITT) for 119 intervention and 122 control group participants with imputed data for missing values (28). Complete case analysis included 101 intervention and 104 control group participants who had outcome measurements for the primary outcome (GWG) available at the end of pregnancy.

Multivariable regression analyses were used to determine the intervention effect on each outcome of interest, adjusting for stratification variables: study site and pre-pregnancy BMI category. Due to a low number of individuals in the underweight category (*n* = 4), these individuals were grouped with normal weight category.

For the primary outcome, GWG within IOM guidelines (yes/no), both a binary regression model and logistic regression model were created. The adjusted odds ratio, 95% CI, and *p* value were computed using a logistic regression model (distribution = binomial, link = logit).

For continuous outcomes, linear regression was performed. Outcomes where the normality assumption was not met were transformed (using natural log [Ln] or square root transformations) before being used in a model. For the remaining binary outcomes, logistic regression analyses were performed. For all models, the results are expressed as an estimate of the adjusted mean difference for continuous outcomes (adjusted odds ratio (OR) for binary outcomes), corresponding two-sided 95% confidence intervals, and associated *p*-values.

For the PrimeScreen dietary assessment scores contrasted between allocation groups over time, generalized estimating equations (GEE) assuming autoregressive (AR(1)) correlation structure were used to account for correlated repeated outcome data within a participant over time. A normal model with identity link function was used. Analysis was controlled for maternal pBMI and study site.

Sensitivity analysis was performed using per protocol analysis and complete case analysis for all outcomes. Individuals were included for per protocol analyses if they had reported gestational weight gain values during their third visit; results were reported based on analysis of imputed data. Individuals included for complete case analysis were based on data available for each outcome and independent variables adjusted in the model; results were reported based on analysis of non-imputed data.

For all subgroup analyses, the treatment effect and confidence intervals for each site and each pre-pregnancy BMI level, along with the interaction *p* value are reported. Forest plots were constructed for the primary outcomes.

All statistical tests were performed using two-sided tests at the 0.05 level of significance. All analyses were performed using SAS software Version 9.4 (SAS Institute Inc., Cary, NC, USA) and SPSS 25 (Chicago, IL, USA). Forest plots were created in Stata software (Version 15.0; College Station, TX, USA)

## 3. Results

### 3.1. Participant Recruitment and Characteristics

**Figure 1:**
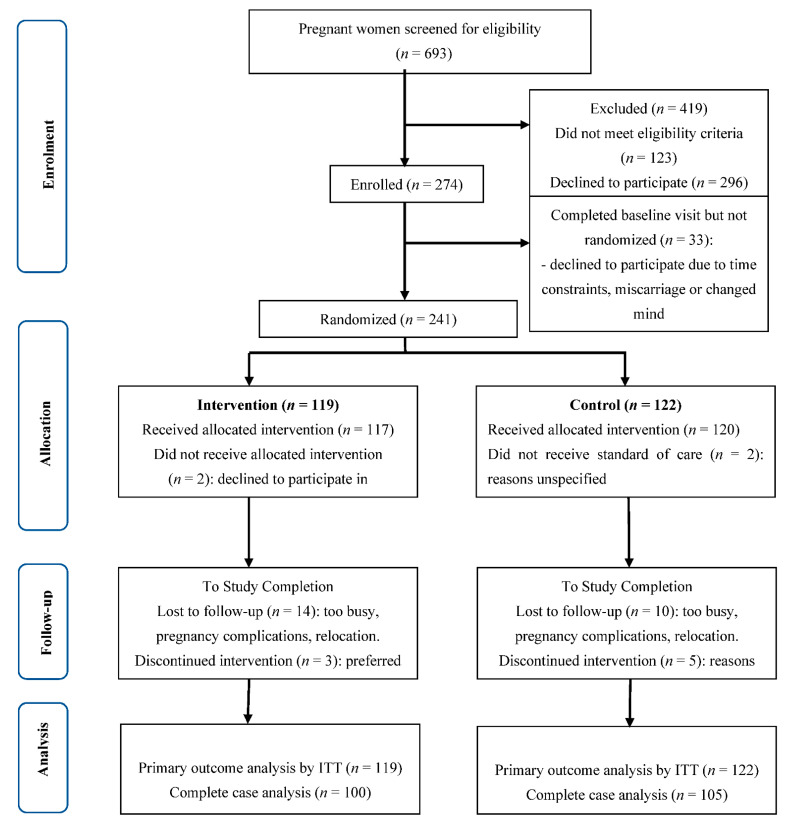
CONSORT diagram for recruitment, randomization, and follow-up of participants.

Recruitment occurred between January 2013 and April 2018 [13] with a total of 693 women screened by phone of whom 123 (17.7%) did not meet eligibility criteria, and 296 (42.7%) declined to participate for reasons noted in Figure 1. Challenges to recruitment of participants in the early stages of the study were detailed in the protocol paper [13]. Of 274 women who consented and attended the first visit, 33 were not randomized because they failed to return for the second visit as noted in Figure 1. After randomization, two in each treatment arm declined to initiate the study; after study initiation, 17 in the intervention group and 15 in the control group withdrew for reasons noted in Figure 1.

At study entry, participant characteristics were well balanced by randomized groups with the majority being of European descent, university educated, family income above $75,000 Canadian, and married/common law (Table 1). About 30% were categorized by pBMI as overweight and about 18% as obese (Table 1).

### 3.2. Gestational Weight Gain (Primary Outcome)

**Figure 2:**
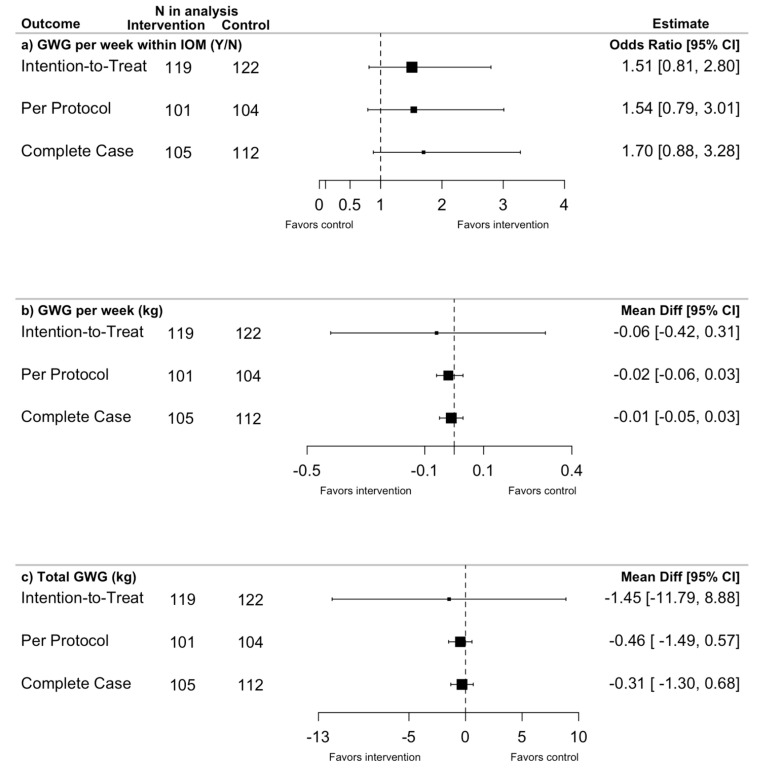
Forest plots of intervention effect on primary outcomes related to gestational weight (GWG) by intention-to-treat, per protocol and complete case analysis adjusted for study site and maternal pre-pregnancy BMI category.

The odds of achieving GWG within the IOM guidelines was 51% higher in the intervention compared to control group by intention-to-treat (ITT) analysis, but this was not statistically significant (OR = 1.51; 95% CI: 0.81, 2.80) (Figure 2). This observation was robust across the per protocol analysis (OR = 1.54; 95% CI: 0.79, 3.01) and the complete case analysis (OR = 1.70; 95% CI: 0.88, 3.28) (Figure 2). Gestational weight gain within the IOM guidelines was achieved by 31% (33/105) of women in the intervention group and 22% (25/112) in the control group. Mean GWG per week was similar between allocation groups (ITT: Mean difference (MD) = −0.06, 95% CI: −0.42, 0.31 kg/week) and was robust across analyses (Figure 2). The total GWG (ITT: MD = −1.45; 95% CI: −1.79, 8.88 kg) was lower but not significantly different in the intervention compared to the control group (Figure 2).

Pre-specified sensitivity analysis conducted for the primary outcome of GWG on sub-groups of BMI found no significant interaction effect between the pBMI category and treatment group (Table 2). In the normal BMI category, about 50% of the intervention and 34% of the control group had GWG within the IOM guidelines (Table 2). In the overweight and obese BMI categories, only 3 to 16% of women met GWG recommendations (Table 2). Both GWG per week and total GWG were similar between intervention and control groups within BMI categories (Table 2). However, only the normal weight BMI category had average weekly and total GWG within the IOM recommended range. Mean weekly GWG was up to double that recommended in overweight and obese BMI categories (Table 2). For mean total GWG, the overweight group exceeded the recommended range only in the control group while in the obese BMI category, the GWG was at or exceeded the upper range of the IOM recommendations for both treatment groups (Table 2).

### 3.3. Pre-Specified Pregnancy and Birth Outcomes

**Figure 3:**
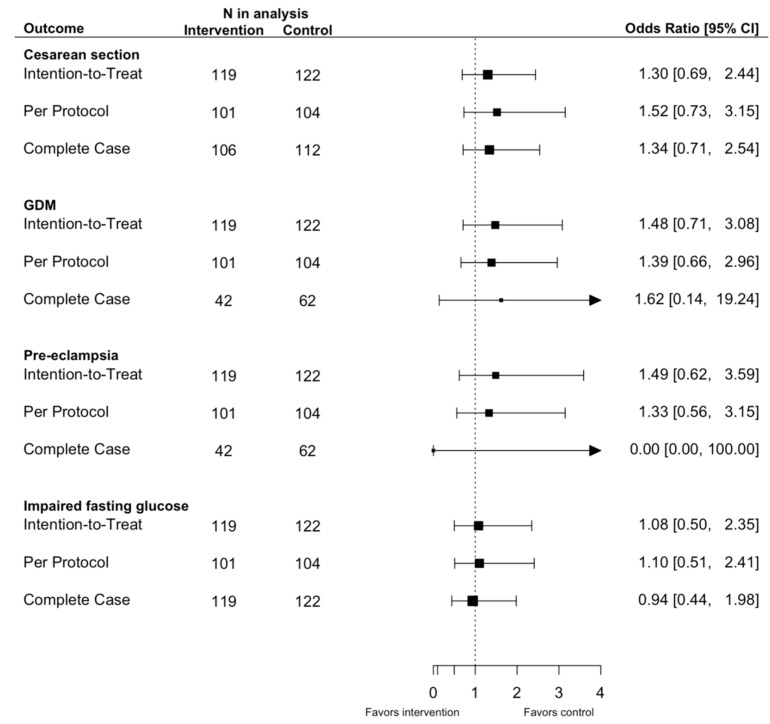
Forest plots of intervention effect for binary pre-specified pregnancy outcomes by intention-to-treat, per protocol, and complete case analysis adjusted for study site and maternal pre-pregnancy BMI category. OR = odds ratio; CI = confidence interval All analyses adjusted for site (Hamilton, London) and pre-pregnancy BMI (preBMI) categories: underweight to normal weight (≤24.9), overweight (25.0–29.9), obese (≥30). Due to a very low number of events, adjusted regression analyses could not be performed on operative vaginal delivery.

Outcomes of Cesarean section, pre-eclampsia, and gestational diabetes (GDM) were more frequent in the control than intervention group but the differences were not statistically significant (Figure 3). Regression analysis could not be performed on all outcomes due to insufficient events of GDM (<5% participants) and pre-eclampsia (<2% participants) due in part to missing data not recorded on the pregnancy birth record. Impaired fasting glucose (defined as blood glucose > 5.1 mmol/L) was similar between treatment groups (Figure 3) and occurred in about 14% of women in both groups.

No intervention effect was observed for mean difference in birth weight (MD = 138 g; 95% CI: −218, 494 g) or gestational age (MD = 0.26; 95% CI: −1.27, 1.80 week) (Table 3). Most (82–87%) infants had birth weights appropriate for gestational age (10th–90th percentile for weight), <8% were small for gestational age (<10th percentile) and <10% were large for gestational age (>90% percentile) (Table 3). Only five infants were born prematurely, two in the intervention group and three in the standard care group.

### 3.4. Pre-Specified Maternal Health Outcomes

At baseline measures of adiposity, blood pressure, fasting glucose, blood lipid profiles, and leptin, insulin, and adiponectin were similar between allocation groups (Table 1). At 36–38 weeks gestation blood pressure was similar between groups (Table 4). Hypertension, defined as SBP > 140 mmHg and DBP > 90 mmHg [24], was observed in 2 women in the intervention and 2 women in the control group at late pregnancy. No intervention effect was observed for maternal depression score at the end of pregnancy (Table 4). At late pregnancy, after about 23–25 weeks of intervention, the mean difference for both sum of skinfolds (MD = 1.5 mm; 95% CI: −31.02, 34.11 mm) and % body fat by BIA (MD = 0.25%; 95% CI: −0.65, 1.15%) were similar between groups (Table 4).

Metabolic profiles including blood glucose, HDL, LDL, total cholesterol, triglycerides, leptin, insulin, adiponectin, and CRP were similar between groups at 36–38 weeks gestation by ITT analysis (Table 5). By complete case analysis, the intervention compared to control group had significantly lower LDL (MD = −0.31 mmol/L; 95%CI: −0.57, −0.06) and total cholesterol (MD = −0.41; 95% CI: −0.72, −0.09 mmol/L), and higher CRP (MD = 0.21; 95% CI: 0.01, 0.42 mg/L) (Table 5).

### 3.5. Dietary and Physical Activity Measures

**Figure 4:**
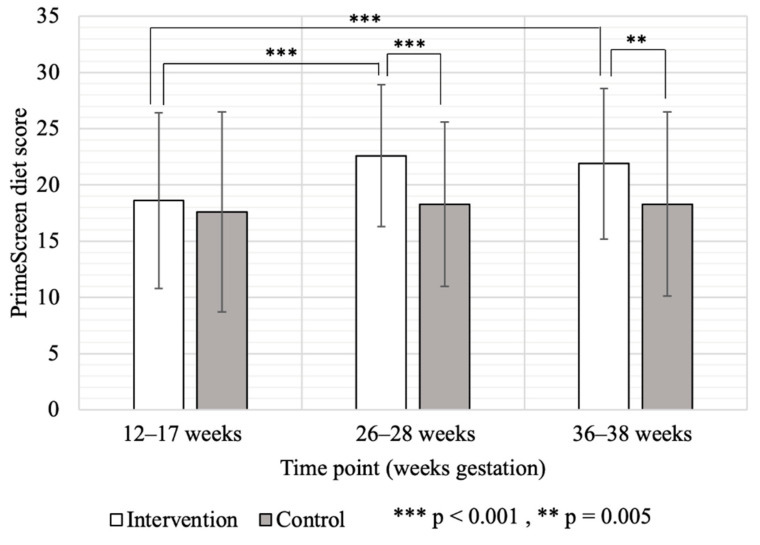
Participant PrimeScreen healthy dietary practice scores in Intervention (*n* = 69) and Control (*n* = 67) groups.

At baseline, diet scores based on PrimeScreen FFQ were similar between intervention and control groups (mean ± SD; 18.7 ± 7.6 1 vs. 7.1 ± 8.7 (Figure 4)). In the intervention group, diet scores improved significantly (*p* < 0.001) over baseline at 26–28 weeks gestation (22.9 ± 6.1) and remained higher at the end of pregnancy (22.5 ± 6.9) (Figure 4) while scores for the control group did not change (18.7 ± 7.7 and 18.7 ± 8.5) (Figure 4). Based on 3-day food records, mean protein intake prior to randomization was similar between groups (Table 6) but rose by design in the intervention group by 28% and was significantly higher than the control group at both 26–28 weeks (MD = 15.0; 95% CI [8.1, 21.9] g/day) and 36–38 weeks (MD = 15.2; 95% CI: 9.4, 21.1 g/day) gestation (Table 7). Total energy intake (~2160 kcal/day) was similar between treatment groups (Table 7) and across pregnancy (Table 6).

Based on accelerometry data, average daily step counts were similar between intervention and control groups throughout pregnancy (Table 6 and Table 7) reducing to ~5550 steps/day at late pregnancy (Table 6). The 10,000 step goal was achieved by only 9.1% of intervention participants at 26–28 weeks gestation and 5.5% of participants at 36–38 weeks gestation. Both groups spent about 50 min per day at baseline in moderate to vigorous physical activity, but this declined to 38 min at the end of pregnancy (data not shown). The total energy expenditure was similar between treatment groups (Table 7) and across pregnancy from 12–17 to 36–38 weeks gestation averaging about 2100 kcal/day (Table 6).

## 4. Discussion

The BHIP structured and monitored diet with at least biweekly nutrition counselling resulted in improvements in overall diet quality, higher protein intake (106 vs. 88 g/day), and stable energy intake across pregnancy. The positive dietary response was not mirrored in achieving the exercise goal of 10,000 steps which proved challenging. Mean daily step counts remained similar across pregnancy between treatment groups and declined from baseline to 36–38 weeks gestation with fewer than 10% of women achieving the target step count despite continued encouragement and walking sessions accompanied by research staff at the bi-weekly visits for the intervention group. Taken together, the observed changes in nutritional practice in the intervention group were likely the explanation for the observed 51% greater chance of achieving a GWG within the current guidelines of the IOM [1] and a mean lower total GWG of 1.45 kg compared to the control, although the differences were not statistically significant.

Sensitivity analysis provided insights into the variation in outcome responses across BMI categories. The normal weight BMI category had a higher achievement of GWG within recommendations (49.2%) than overweight (10.3%) or obese (5.9%) women. This parallels the finding of a RCT of a behavioral intervention in women (*n* = 358) from all BMI categories wherein a significant main effect for BMI category for excessive GWG was observed compared with all other weight-gain categories [28].

Cardiometabolic monitoring of blood glucose, blood pressure, and lipid profiles showed similarity between intervention and control group and few abnormalities. Only 14% of women developed impaired fasting glucose, and few (*n* = 4) developed hypertension. In the complete case analysis, fasted blood LDL, total cholesterol, and CRP were significantly lower in the intervention group but not likely of clinical significance.

For infants, birth outcomes did not differ by treatment group. Birth weights were appropriate for gestational age with only 6–10% in the >90th percentile range and 5–6% in the <10th percentile range.

In interpreting our findings compared to other recent similar clinical trials, we observed that the proportion of women meeting the IOM GWG recommendations in the BHIP study were higher than the prospective meta-analysis of 7 harmonized RCTs called LIFE-Moms in the US in which only 17.6% in the intervention (compared to 33% in BHIP) and 10.8% in the standard care group (compared to 22% in BHIP) attained GWG within the IOM recommendations despite having a significant intervention effect in lower total weight gain [29]. Further, the 1.45 kg total lower GWG in the intervention group was twice that observed (−0.7 kg) for the intervention group reported in the individual participant data (IPD) meta-analysis of diet and physical activity-based interventions [30] and similar to the LIFE-Moms RCT of −1.58 kg [29]. The observed −1.45 kg lower GWG in our intervention group represents a >10% difference of the expected gain using the IOM recommendation of 11–16 kg for the normal weight BMI category. This could be considered a clinically significant difference.

That the BHIP intervention did not impact maternal pregnancy outcomes is in line with previous meta analyses of RCTs that found a treatment effect on GWG but not for GDM, preeclampsia, or pregnancy-induced hypertension [31] and is also consistent with prenatal lifestyle intervention studies in the United States [32] and Australia [33] and the recent IPD-meta-analysis that showed a significant reduction in Cesarean section with the intervention but not for GDM, hypertensive disorders, or preterm delivery [34]. The low rates of GDM and pre-eclampsia may reflect the general good health of our study groups. The infant birth outcomes in the BHIP study were also consistent with the majority of previous meta-analysis reviews of diet and exercise interventions wherein no significant differences were reported in infant birthweight, macrosomia, or prevalence estimates of SGA and LGA infants [29,32,33,35], as well as a UK study in obese pregnant women in the United Kingdom (*n* = 1555) [36] and in women of all BMI categories in Brazil (*n* = 639) [37].

The strategy of a multi-component intervention in pregnancy for GWG management as designed in BHIP aligns with recommendations of a recent evidence review [38]. Further, this study addressed the identified under-reporting of intervention content or “active intervention ingredients” for nutrition and exercise interventions by specific adherence measures [39]. The improved diet quality score and higher protein intake indicated adherence to the structured and individualized dietary guidance with counselling in the intervention group. The frequency of knowledge sharing and individualized guided support from the study nutritionist, including a nutrition handbook, in addition to the provision of self-selected low-fat dairy foods, were likely other motivating factors to improve dietary practices in the intervention group.

Our results on physical activity measures align with a recent multi-component RCT in which a goal of 10,000 steps per day was not attained out to late pregnancy [40]. Current evidence-based Canadian pregnancy guidelines for physical activity during pregnancy recommend at least 150 min of moderate-intensity exercise a week such as brisk walking, accumulated over a minimum of three days, but do not specify a daily step count [41]. Since BHIP participants averaged 50 min/day of moderate to vigorous physical activity at 26–28 weeks gestation and 38 min/day at 36–38 weeks gestation, the recommended goal of 150 weekly minutes of physical activity for health benefits [40] would have been achieved over 3 days in mid and 4 days in late pregnancy. The amount of time spent being physically active by BHIP participants fell within recommendations in a recent evidence review [38]. Maintaining 10,000 daily steps may be an unreasonable goal, particularly in pregnancy; and it has been questioned for use even in the non-pregnant population [42]. Many barriers to achieving exercise recommendations for pregnancy include time limitations [43,44,45], pregnancy-specific complications [44,45], or the misconception that exercise will cause harm to their offspring [46,47].

A major strength of the BHIP randomized trial was that it fulfilled many of the recently recommended attributes of a multi-component approach to influencing GWG [38] and addressed several methodological issues in studies on GWG noted to limit the strength of evidence in a recent National Academy of Medicine discussion paper [3]. Examples include initiation in early pregnancy; supervised physical activity; individualized intervention of energy intake and diet plan with counselling; and GWG monitoring. Further, the two diet assessment tools and accelerometry for quantitative physical activity measures employed rather than questionnaires provided measures of fidelity and adherence to the prescribed goals in the intervention group and comparison between groups. The BHIP study was also conducted in a community setting and included all pBMI categories (except extreme obesity), whereas many previous studies were targeted only to women with pregravid obesity and/or overweight recruited through hospital clinics.

Limitations of the study relate to generalizability of the findings. The recruitment sites were mostly primary care clinics of midwives or family doctors, and eligibility criteria excluded women with limited comprehension of the English, with documented signs of depression or a pre-pregnancy BMI above 40 kg/m^2^. Our study population was mostly Caucasian, holding a university degree, and a medium to high socio-economic status, thus being representative of populations in the cities where recruitment occurred as they are modern urban centers with universities, colleges, and major commerce. Finally, due to funding constraints, we were not able to achieve the original sample size.

## 5. Conclusions

While the structured and monitored nutrition and exercise intervention did not result in a statistically significantly greater proportion of women achieving recommended GWG compared to usual prenatal care offered in our community, the observed lower GWG of 1.45 kg in the intervention groups aligns with previous studies and may be clinically significant. Improvements in dietary practices within the intervention group did lead to healthier dietary practice scores and a higher protein intake as recently recommended [48]. The elements of the BHIP approach to improving nutrition may inform future studies of nutrition in pregnancy; and the targets for nutrient intake such as energy for normal weight, overweight, and obese women may need to be different [3]. While the exercise goal was not attained, the average step counts and intensity observed were similar to other studies [40], were within current guidelines [37], and were not associated with high blood pressure. Perhaps the attained step counts reveal the limitations of capacity to exercise in busy women during pregnancy and serve as realistic goals. Taken together, our results can inform future guidance for feasible guidance on nutrition and physical activity in pregnancy. As shown in the BHIP and other studies cited, achieving the current IOM recommendations for GWG [1] (now over 12 years old) is not easily attainable for many pregnant women. Our study demonstrated that a higher than recommended GWG was not associated with a preponderance of adverse pregnancy or birth outcomes suggesting it might be timely to re-visit the recommendations for ideal gestational weight gain.

## Figures and Tables

**Table 1:** Demographic characteristics of participants at 12–17 week gestation. Values are raw data.

Demographic Characteristic	Intervention (*n* = 119)	Control (*n* = 122)
	**Mean (SD) ^1^**	**Mean (SD)**
Maternal age (year)	31.6 (3.9)	31.3 (4.3)
Gestational age at randomization (week)	13.75 (1.75)	13.60 (1.61)
Maternal weight, kg	72.4 (13.4)	71.8 (13.1)
Pre-pregnancy BMI ^2^ (kg/m^2^)	25.7 (4.5)	25.3 (4.6)
	***n* (%)**	***n* (%)**
Pre-pregnancy BMI (kg/m^2^) category		
Underweight (<18.5)	2 (1.7)	2 (1.6)
Normal weight (18.5–24.9)	61 (51.3)	62 (50.8)
Overweight (25.0–29.9)	34 (28.6)	37 (30.3)
Obese (30.0–39.9)	22 (18.5)	21 (17.2)
University education	114 (99.1)	114 (95.8)
Missing	4	3
Race/ethnicity		
European descent	105 (88.2)	107 (87.7)
Mixed/other	14 (11.8)	13 (10.7)
Missing	0	2
Total family income		
<$45,000	7 (5.9)	10 (8.9)
$45,000–$74,999	19 (16.2)	25 (22.1)
>$75,000	91 (77.8)	78 (69.0)
Missing (=Unknown)	2	9
Married/living with significant other	117 (98.3)	114 (96.6)
Missing	0	4
Nulliparous	58 (49.15)	56 (47.46)
Missing	1	4
Study site (city)		
Western U (London)	38 (31.9)	39 (32.0)
McMaster U (Hamilton/Burlington)	81 (68.1)	83 (68.0)
**Physical and Metabolic Measures**	**Mean (SD)**	**Mean (SD)**
Maternal fat mass		
Bioelectrical impedence (BIA), % body fat	33.98 (6.55)	34.11 (7.14)
Missing	8	3
Sum of skinfolds (SFT) (mm)	72.28 (23.32)	72.07 (26.15)
Missing	3	4
Maternal blood pressure (mmHg)		
Systolic	109.20 (10.43)	108.95 (10.49)
Missing	2	3
Diastolic	68.96 (7.54)	69.24 (7.69)
Missing	2	3
Maternal physical activity		
Energy expenditure (kcal/day)	2085 (312)	2084 (356)
Missing	11	11
Average daily step count	7043 (2655)	6587 (2545)
Missing	11	11
Maternal depression score	4.34 (3.13)	4.24 (2.79)
Missing	1	1
Maternal metabolic outcomes		
Fasting glucose (mmol/L)	4.84 (0.60)	4.77 (0.47)
Missing	13	10
Triglycerides (mmol/L)	1.29 (0.60)	1.28 (0.42)
Missing	13	10
Total cholesterol (mmol/L)	5.42 (1.18)	5.24 (0.97)
Missing	13	10
HDL-cholesterol (mmol/L)	1.86 (0.44)	1.81 (0.39)
Missing	13	10
LDL-cholesterol (mmol/L)	2.97 (0.81)	2.85 (0.71)
Missing	14	10
Leptin (ng/mL)	31.76 (25.31)	30.14 (25.54)
Missing	9	7
Insulin (pmol/L)	42.43 (34.81)	40.88 (29.55)
Missing	10	8
Adiponectin (µg/mL)	8.19 (3.17)	8.45 (4.10)
Missing	9	7
CRP (mg/L)	6.29 (4.86)	6.21 (5.65)
Missing	9	8

^1^ SD = Standard Deviation; ^2^ BMI = Body Mass Index.

**Table 2:** Gestational weight gain (GWG) outcomes in intervention compared to control group across pre-pregnancy body mass index (pBMI) categories (intention-to-treat sensitivity analysis).

	Intervention (*n* = 119) ^1^ % Participants	Control (*n* = 122) ^1^ % Participants	Intervention Effect		Interaction *p*
			OR ^2^ (95% CI) ^3^	Mean Difference (95% CI) ^3^	
Within IOM guidelines for GWG/week					
Normal Weight ^4^	49.2%	34.4%	1.92 (0.93, 4.00)		.
Overweight ^4^	10.3%	3.1%	1.38 (0.23, 8.27)		0.739
Obese ^4^	5.9%	15.8%	0.26 (0.02, 2.74)		0.110
**GWG per week (kg/week) ^5^**	**Mean (SD)**	**Mean (SD)**			
Normal Weight ^4^ IOM recommendation (0.42 kg/week)	*n* = 58 0.48 (0.15)	*n* = 61 0.49 (0.16)		−0.03 (−0.08, 0.02)	.
Overweight ^4^ IOM recommendation (0.28 kg/week)	*n* = 30 0.48 (0.13)	*n* = 32 0.53 (0.15)		−0.01 (−0.07, 0.07)	0.962
Obese ^4^ IOM recommendation (0.22 kg/week)	*n* = 17 0.45 (0.16)	*n* = 19 0.38 (0.22)		−0.22 (−2.25, 1.81)	0.813
**Total GWG (kg)**					
Normal Weight ^4^ IOM recommendation (11.5–16.0 kg)	*n* = 58 11.02 (3.40)	*n* = 61 11.43 (3.67)		−0.69 (−1.94, 0.55)	.
Overweight ^4^ IOM recommendation (7.0–11.5 kg)	*n* = 30 11.30 (3.11)	*n* = 32 12.22 (3.69)		−0.33 (−2.02, 1.36)	0.764
Obese ^4^ IOM recommendation (5.0–9.0 kg)	*n* = 17 10.02 (3.67)	*n* = 19 8.92 (5.34)		−0.40 (−3.60, 4.41)	0.539

^1^ 14 values missing from intervention; 10 missing from control. ^2^ OR = odds ratio. ^3^ CI = confidence interval. ^4^ Underweight to normal weight (≤24.9), overweight (25.0–29.9), obese (≥30.0–39.9). Underweight and normal weight were grouped together due to a low number of observations in the underweight category (*n* = 4). ^5^ Calculated over the second and third trimesters of pregnancy. All analyses adjusted for study site (Hamilton, London) but not PBMI as the analysis was for prevention effect across pBMI categories.

**Table 3:** Pre-specified infant birth outcomes at birth in intervention compared to control groups by intention-to-treat (ITT) analysis. Values for intervention and control are mean (SD) of raw data with missing values noted.

	Intervention *n* = 119	Control *n* = 122	Intervention Effect by ITT Mean Difference (95%CI) ^1^	*p*
Birth weight (g)	3553 (508)	3476 (456)	138.10 (−217.72, 493.93)	0.432
Missing	12	11		
Gestational age (weeks)	39.76 (1.30)	39.72 (1.29)	0.26 (−1.27, 1.80)	0.718
Missing	13	10		
Birth weight category, *n* (%) ^2^				
Small for gestational age < 10%	8 (7.55)	7 (6.31)	^2^	
Appropriate for gestational age 10–90%	87 (82.08)	97 (87.39)		
Large for gestational age > 90%	11 (10.38)	7 (6.31)		
Missing	13	11		
Birth trauma, *n* (%)	3 (2.91)	0	^2^	
Missing	16	16		
5-min Apgar score	8.92 (0.05)	8.93 (0.40)	^2^	
Missing	17	16		

^1^ OR = odds ratio; CI = confidence interval. ^2^ Due to a low number of events, adjusted regression analyses could not be performed on the categorical outcomes of SGA/AGA/LGA, birth trauma, and 5-min Apgar score. All analyses adjusted for site (Hamilton/Burlington, London) and pre-pregnancy BMI categories: underweight to normal weight (BMI ≤ 24.9), overweight (BMI = 25.0 – 29.9), obese (BMI = 30.0 – 39.9).

**Table 4:** Pre-specified maternal health outcomes at 36–38 weeks gestation in intervention compared to control groups. Values are mean (SD) unless otherwise noted.

Outcome	Intervention (*n* = 119) *n* (%)	Control (*n* = 122) *n* (%)	Intervention Effect			
			Complete Case Analysis		ITT Analysis	
			Mean Difference (95% CI) ^1^	*p*	Mean Difference (95% CI)	*p*
Maternal Fat Mass						
% body fat by BIA	38.1 (6.1)	37.7 (5.9)	0.09 (−0.76, 0.95)	0.829	0.25 (−0.65, 1.15)	0.582
Missing	24	26	55		−	
Sum of Skinfolds (mm)	77.1 (22.9)	75.8 (24.3)	−1.07 (4.86, 2.72)	0.579	1.55 (−31.01, 34.11)	0.919
Missing	22	26	49		−	
Maternal Blood Pressure (mmHg)						
Systolic	113 (12)	113 (11)	−0.40 (−3.28, 2.48)	0.784	−2.43 (−11.78, 6.91)	0.593
Missing	22	26	49		−	
Diastolic	73 (9)	74 (9)	−1.17 (−3.38, 1.05)	0.300	−2.64 (−8.55, 3.26)	0.371
Missing	22	26	49			
Maternal depression score, median	3.0 (1,5)	3.0 (1,6)	−0.06 (−0.30, 0.18) ^2^	0.609	−0.10 (−0.32, 0.13) ^2^	0.400
Missing	23	25	50		−	

^1^ CI = confidence interval. ^2^ Regression analysis based on square root transformation of outcome. All analyses adjusted for site (Hamilton/Burlington, London) and pre-pregnancy BMI categories: underweight to normal weight (BMI ≤ 24.9), overweight (BMI = 25.0–29.9), obese (BMI ≥ 30.0–39.9).

**Table 5:** Pre-specified maternal metabolic profiles at 36–38 weeks gestation for intervention and control groups based on fasting blood samples.

	Group Mean (SD) Median (Q1, Q3) ^1^		Intervention Effect			
			Complete Case Analysis		ITT Analysis	
Metabolite	Intervention *n* = 119	Control *n* = 122	Mean Difference (95% CI)	*p*	Mean Difference (95% CI)	*p*
Glucose, mmol/L	4.6 (0.5)	4.6 (0.5)	−0.04 (−0.17, 0.08)	0.481	−0.02 (−0.16, 0.12)	0.787
Missing	31	26	61			
HDL, mmol/L	1.88 (0.44)	1.87 (0.46)	−0.03 (−0.12, 0.07)	0.601	−0.02 (−0.12, 0.09)	0.759
Missing	30	28	62			
LDL, mmol/L	4.00 (1.11)	4.17 (1.09)	−0.31 (−0.57, −0.06)	0.015	−0.24 (−0.50, 0.02)	0.073
Missing	31	30	66			
Triglycerides, mmol/L	2.53 (0.80)	2.66 (0.79)	−0.18 (−0.36, 0.01)	0.062	−0.16 (−0.38, 0.07)	0.172
Missing	30	28	62			
Total cholesterol, mmol/L	7.03 (1.38)	7.26 (1.33)	−0.41 (−0.72, −0.09)	0.012	−0.29 (−0.62, 0.05)	0.096
Missing	30	28	62			
Insulin, pmol/L	52.08 (34.06, 82.36)	47.95 (32.16, 74.31)	<0.01 (−0.15, 0.15) ^2^	0.999	−0.04 (−0.21, 0.13) ^2^	0.633
Missing	27	25	56			
Leptin, ng/mL	29.83 (15.97, 49.35)	30.32 (15.47, 54.63)	−1.37 (−6.67, 3.92)	0.609	−0.94 (−8.56, 6.68) ^2^	0.805
Missing	26	25	54			
Adiponectin, ug/mL	6.97 (2.62)	7.32 (3.21)	0.02 (−0.55, 0.58)	0.956	−0.05 (−0.97, 0.87)	0.915
Missing	26	25	54			
CRP, mg/L	4.29 (2.39, 7.37)	4.53 (1.92, 7.36)	0.21 (<0.01, 0.42) ^2^	0.046	−0.11 (−0.33, 0.55) ^2^	0.600
Missing	26	25	55			

^1^ Descriptive statistics for continuous outcome I are presented using medians and quartiles (Q1 and Q3). ^2^ Regression analysis based on natural log (ln) transformation of outcome. All analyses adjusted for site (Hamilton, London) and pre-pregnancy BMI categories: underweight to normal weight (BMI ≤ 24.9), overweight (BMI = 25.0–29.9), obese (BMI = 30.9). Underweight and normal weight were grouped together due to a low number of observations in the underweight category (*n* = 4).

**Table 6:** Macronutrient intake and physical activity measures at 3 timepoints across pregnancy.

Gestation	12–17 Weeks		26–28 Weeks		36–38 Weeks	
Group	Intervention	Control	Intervention	Control	Intervention	Control
	Mean (SD)		Mean (SD)		Mean (SD)	
	*n* = 115	*n* = 117	*n* = 84	*n* = 91	*n* = 91	*n* = 89
Diet/Physical Activity Measure						
3-day Food Record Data						
Energy intake; kcal/day	2148 ^1^ (491)	2149 (539)	2195 (423)	2129 (499)	2203 (545)	2152 (496)
Protein intake; g/day	84.6 (22.5)	87.5 (23.8)	108.6 (26.8)	87.9 (23.2)	104.4 (25.4)	87.4 (23.2)
Body Media Data						
Energy Expenditure; kcal/day	2085 (312)	2084 (357)	2152 (338)	2127 (387)	2185 (375)	2157 (325)
Daily step count	7043 (2655)	6588 (2545)	6938 (2717)	6337 (2647)	5538 (2490)	5563 (2208)

^1^ Values are mean (SD) based on raw data of available dietary records. SD = Standard deviation.

**Table 7:** Intervention effect on dietary and physical activity measures at 26–28 weeks and 36–38 weeks of gestation.

Outcome Measure ^1^	Intervention Effect As at 26–28 Week Gestation				Intervention Effect at 36–38 Week Gestation			
	Complete Case Analysis		ITT Analysis		Complete Case Analysis		ITT Analysis	
	Mean Difference (95%CI)	*p*	Mean Difference (95%CI)	*p*	Mean Difference (95% CI) ^1^	*p*	Mean Difference (95% CI)	*p*
Energy Intake, kcal/day	60 (−59, 180)	0.320	28 (−984, 154)	0.662	56 (−83, 195)	0.429	32 (−136, 200)	0.700
Missing	66				61			
Protein Intake, g/day	21.3 (14.4, 28.1)	<0.001	15.0 (8.1, 21.9)	<0.001	19.2 (12.8, 25.5)	<0.001	15.2 (9.4, 21.1)	<0.001
Missing	66				61			
Energy Expenditure, kcal/day ^2^	0.02 (−0.02, 0.06)	0.337	<0.01 (−0.02, 0.02)	0.904	<0.01 (−0.04, 0.04)	0.922	<0.01 (−0.02, 0.02)	0.971
Missing	67				94			
Step count/day	225 (−45, 1901)	0.512	69 (−554, 692)	0.827	−415 (−1056, 226)	0.203	−315 (−900, 271)	0.286
Missing	67				91			

^1^ Values based on 3-day food records and accelerometry measures adjusted for baseline (V1b) and design variables (pre-pregnancy body mass index and site). ^2^ Regression analysis was based on the log transformation of the outcome.

## Data Availability

The datasets used and analyzed for this manuscript can be made available in the future from the corresponding author on reasonable request (Stephanie Atkinson at satkins@mcmaster.ca).
